# Single-domain antibodies and aptamers drive new opportunities for neurodegenerative disease research

**DOI:** 10.3389/fimmu.2024.1426656

**Published:** 2024-08-22

**Authors:** Rachel L. Shoemaker, Roxanne J. Larsen, Peter A. Larsen

**Affiliations:** ^1^ Minnesota Center for Prion Research and Outreach (MNPRO), University of Minnesota, St. Paul, MN, United States; ^2^ Department of Biomedical and Veterinary Sciences, University of Minnesota College of Veterinary Medicine, St. Paul, MN, United States; ^3^ Priogen Corp., St. Paul, MN, United States

**Keywords:** Alzheimer’s disease, nanobody, Parkinson’s disease, prion disease, therapeutics, transmissible spongiform encephalopathy

## Abstract

Neurodegenerative diseases (NDs) in mammals, such as Alzheimer’s disease (AD), Parkinson’s disease (PD), and transmissible spongiform encephalopathies (TSEs), are characterized by the accumulation of misfolded proteins in the central nervous system (CNS). Despite the presence of these pathogenic proteins, the immune response in affected individuals remains notably muted. Traditional immunological strategies, particularly those reliant on monoclonal antibodies (mAbs), face challenges related to tissue penetration, blood-brain barrier (BBB) crossing, and maintaining protein stability. This has led to a burgeoning interest in alternative immunotherapeutic avenues. Notably, single-domain antibodies (or nanobodies) and aptamers have emerged as promising candidates, as their reduced size facilitates high affinity antigen binding and they exhibit superior biophysical stability compared to mAbs. Aptamers, synthetic molecules generated from DNA or RNA ligands, present both rapid production times and cost-effective solutions. Both nanobodies and aptamers exhibit inherent qualities suitable for ND research and therapeutic development. Cross-seeding events must be considered in both traditional and small-molecule-based immunodiagnostic and therapeutic approaches, as well as subsequent neurotoxic impacts and complications beyond protein aggregates. This review delineates the challenges traditional immunological methods pose in ND research and underscores the potential of nanobodies and aptamers in advancing next-generation ND diagnostics and therapeutics.

## 1 Introduction

A defining characteristic across numerous mammalian neurodegenerative diseases (NDs) is the production and subsequent aggregation of misfolded proteins in the central nervous system (CNS). Classic examples include neurofibrillary tangles (NFTs) of the tau protein observed in Alzheimer’s disease (AD) and prion protein fibrils and plaques observed across animal and human transmissible spongiform encephalopathies (TSEs) (1). Despite the pathogenic nature of misfolded proteins, individuals impacted by NDs typically display limited to no immune response. The mammalian adaptive immune system’s reactivity is primarily restricted to non-self or foreign molecules and pathogens, whereas dysfunctional or nonviable proteins from self produce little to no response (2). Insufficient immune activation represents clear challenges when considering antibody-focused ND diagnostics and therapeutics, which require specific binding to target biomarkers and pathogenic proteins. Examples of diagnostic limitations include the lack of specific antemortem immuno-assays for Alzheimer’s Disease (AD), Parkinson’s Disease (PD), and Chronic Traumatic Encephalopathy (CTE) and no widely available antibodies specific to infectious conformations of TSE prions (PrP^Sc^). When considering ND immunotherapeutics using traditional monoclonal antibodies (mAbs), a multitude of challenges persist related to tissue and biophysical specific barriers, including limitations of mAbs in crossing the blood-brain barrier (BBB), deep tissue penetration, and maintenance of biophysical stability. Moreover, intracellular misfolded protein fibrils require drug-delivery systems that can solubilize and revert to their native scaffold.

In light of these challenges, the scientific community is exploring alternative options to mAbs for ND immunodiagnostic and therapeutic applications. Recent developments in single-domain antibody and aptamer research hold great promise for overcoming the limitations of traditional antibodies, and mounting evidence indicates these molecules can be leveraged for both diagnostic and therapeutic ND applications. Single-domain antibodies, or nanobodies, are Ig isoforms that lack light chain sequences, consisting of heavy-chain variable domains (VHH) in camelids and variable new antigen receptors (VNARs) in cartilaginous fishes. Nanobodies are substantially smaller than traditional mAbs (i.e., shark nanobody ~12-15 kD vs. mammalian IgG ~150 kD) and have soluble variable domains (3). A growing body of research has focused on leveraging nanobodies for a variety of applications, including ND diagnostics and therapeutics (4–9). In parallel, encompassing qualities attributed to nanobodies, aptamers are synthetic molecules generated by DNA or RNA ligands with low production time and costs (10, 11). Both molecules have intrinsic properties that are attractive for ND research and clinical applications.

In this review, we first highlight the limitations of traditional immunology-based approaches to ND research. We then introduce nanobodies, aptamers, and the ND-associated misfolded proteins that can be targeted using these molecules to develop next-generation ND diagnostics and therapeutics.

## 2 The challenge of “The Self”: limitations of the mammalian adaptive immune system for pathogenetic protein recognition

The mammalian immune system is a highly complex and proficient network of organs, cells, and molecules with the sole purpose of defending the host from harmful invaders, but what if the invader originates from the host itself? The innate and adaptive mammalian immune systems are highly adept at protecting the host from foreign molecules by generating incredibly diverse antibody repertoires for a vast array of encountered pathogens. There are many ways to activate the innate immune response; for example, the detection of foreign pathogens by antigen-presenting cells (APCs) recruits neutrophils that induce proinflammatory chemokines and cytokines (12, 13). Adaptive immunity is initiated upon antigen uptake of APCs, which then migrate to lymph nodes and present to naive T cells (14). Soluble antigen exposure can induce antibody production from naive B cells, many of which are initially lower affinity immunoglobulin isoforms (i.e., IgM). Further interactions with antigen and activated T cells can induce class switching and somatic hypermutation, resulting in affinity maturation that leads to antibodies with increased antigen binding capabilities (15).

The immune system can leverage a multitude of strategies to fight off both foreign pathogens and damaging or dysfunctional self-molecules, such as pathogen-associated molecular patterns (PAMPs) and danger-associated molecular patterns (DAMPs), respectively (16). However, there are several scenarios in which immune response pathways fail to protect the host. A portion of the immune system focuses on eliminating self-reactive lymphocytes to prevent autoimmunity (17). Various factors can induce autoimmunity, including inflammation, genetics, environmental exposure, and apoptosis. Chronic inflammation and apoptosis can result from cellular or proteomic dysfunction associated with cancer and NDs, where the enhanced detection of DAMPs induces overreactivity to autoantigens (18). This overreactivity or hypersensitivity to autoantigens can limit the ability to detect and respond to the actual problem while facilitating disease progression. Self-tolerant individuals with increased exposure to DAMPs from NDs go vastly undetected, leading to prolonged pre-clinical periods without symptom presentation while also facilitating disease progression (19).

The protein-only hypothesis of prion disease, proposed by Dr. Stanley Prusiner in 1982, was a fundamental departure from traditional infectious disease biology, postulating infectious proteins (i.e., prions), not bacterial or viral agents, could cause disease (20). Most NDs are classified as prion or prion-like diseases, with neuropathogenic features arising from cascading protein-misfolding events. Prion diseases (i.e., BSE, CJD, scrapie, etc.) flout biological norms, with identical amino acid sequences between healthy and infectious protein conformations that thwart any sustained or specific immune response against PrP^Sc^. Notably, a humoral immune response to misfolded proteins is mainly absent for many NDs, especially those of the CNS (21, 22). This is not surprising, given that most self-reactive B and T lymphocytes are eliminated before exiting to the periphery, and those that do cannot recognize misfolded proteins, given their tendency to be insoluble (23). B cell receptors may recognize and bind novel PrP^Sc^ epitopes due to their ability to recognize both linear and discontinuous antigenic epitopes. However, this initial B cell response would be muted due to the inability to break down insoluble proteins for presentation on MHCII to facilitate CD4+ T cell help (24). In addition, APCs might uptake misfolded prion proteins but cannot digest infectious variants that form a protease-resistant hydrophobic core (25). If these proteins cannot be digested, they cannot be presented on MHC molecules, leading to a lack of T cell activation and subsequent help for high-affinity antibody production (26–30). Moreover, accumulating misfolded proteins can pose problems by blocking pathways in APCs, impeding or preventing their ability to perform regular tasks.

Another challenge of the mammalian immune system’s response to ND is the sheer size of traditional antibodies (i.e., ~ 150 to 950 kD for IgG and IgM) (31). Despite the immune system’s difficulty in producing high-affinity mAbs specific to ND proteins, there are methods and animal models to overcome these hurdles for use in ND diagnostics and therapeutics. However, the size and insolubility of mAbs prohibit transcytosis, a process where molecules are endocytosed and deposited into a new surface across the plasma membrane, limiting deep-tissue penetration and passage into the brain (32–34). For passage across the BBB, mAbs are restricted to specific Fcɣ or neonatal Fc receptors, limiting the amount of mAbs delivered to the brain and correlate with both the low percentage of mAb drug delivery and extremely high costs (35, 36). More challenges are presented as most infectious proteins form a tightly bound hydrophobic core while retaining identical amino acid sequences to their healthy conformation, creating specificity limitations in mAbs (37). Consequently, mAbs that bind to specific yet identical sites within the functional and dysfunctional self-proteins cannot discern between the healthy and infectious protein isoforms (38). While this underscores limitations in the mammalian immune response and effective therapeutics, it also has encouraged unconventional immunodiagnostic and therapeutic solutions.

Physiological barrier structures in most animals are complex and difficult to surpass for a reason, particularly the blood-brain barrier. Effective drug delivery to the brain has more challenges than size for passing the BBB itself. Once past the barrier, many factors must be considered for a successful therapeutic (39). Researchers have experienced limitations related to vesicles for intracellular transport, stability in pH and electrical charge changes, renal clearance rate, and more (40–42). For the last 50 years, immunology and engineering research has tried to overcome size, specificity, and biophysical stability concerns by producing fragments with solely the antigen-binding region. However, doing so significantly reduces stability and affinity (43–45). Strategies to produce ND-specific mAbs mainly require knock-out bioassay models, which can be useful but costly (46, 47). The production cost of mAbs themselves after identification and isolation is exceedingly high, limiting accessibility for research, diagnostics, and therapeutics (3, 48). Moreover, mAb biophysical stability varies in mammalian or monoclonal models, impacting its specificity and affinity, resulting in a loss of reliability in diagnostics and treatments (49, 50). BBB breakdown has been well recorded in nearly all NDs, resulting in microhemorrhages from endothelial damage and capillary leakage, impaired glucose transport and drug regulation, and immune cell infiltration (51–53). There is an increasing need to address other neuropathological hallmarks of ND, like massive neuronal loss and neuroinflammation; therapeutics not sensitive to this have a higher probability of causing further damage (54–56). Moreover, pre-clinical studies lack endpoints specific to immunotoxicity, imperative safety aspects, and limitations of animal models for predicting toxicity (57, 58).

Despite the observations above, other studies have shown aspects of the immune system that restrict and abet prion replication (59). In diseases such as Chronic Wasting Disease (CWD) in North American cervids, early prion replication within lymphoid tissues implicates a significant role of the lymphoreticular system (60, 61). Moreover, proteins in some NDs begin as soluble molecules, like amyloid-beta monomers, with recent findings showing a correlation between the CNS and the adaptive immune response (62–65). Another group discovered memory B cells displaying IgG reactivity to the disease-associated hyperphosphorylated isoform of tau (66). These findings suggest that the immune system might play a more significant role in NDs than originally hypothesized (67).

## 3 Single-domain antibodies

In the 1990s, a new immunogenic class of variable-heavy-chain-only antibodies (VHH) was discovered in camelids, opening the door to specific, high-affinity single-domain binding regions called nanobodies (Nbs) (68). The absence of the first constant domain within the heavy and light chains results in a smaller molecular mass than mAbs of around 80-95 kDa (69). Camelid VHH Nbs consists of two constant domains, a hinge region and a variable heavy chain domain, with antigen-binding capabilities that do not require domain pairing (70, 71). VHHs have highly conserved variable domain regions called complementarity-determining regions (CDR), the central entity involved with antigen binding (72). Predominant binding in the CDR3 region enables epitope binding in regions unreachable or concealed to mAbs.

Another heavy-chain-only new antigen receptor Ig subtype, variable new antigen receptors (VNARs), was discovered in cartilaginous fishes (73). VNARs are composed of two highly soluble variable domains that mediate antigen-binding interactions. Each independent variable domain is around 12 kDa, significantly smaller than mAbs (74, 75). This characteristic, along with their intrinsic stability, solubility, and high affinity for their targets, makes VNARs coveted for diagnostics and therapeutics and have coined a “silver bullet” reputation (3). VHHs and VNARs can withstand extreme temperatures, pH, alkaline and enzymatic environments, and pressure, making them suitable for novel therapeutic applications (71, 76). Moreover, cartilaginous fishes have one of the oldest adaptive immune systems in the world and lack many highly conserved mammalian immunity genes. For example, sharks can produce robust immune responses upon immunization of the natively folded or misfolded mammalian proteins associated with neurodegeneration (77). Multiple immunizations over four months induce a secondary immune response and affinity maturation, producing highly specific VNARs (78, 79).

Nanobody research has resulted in significant progress in the areas where mAbs are limited; however, restricted access to the animal models and potential immunotoxic effects of a non-human model has halted progress on the utility of nanobodies for ND research (3, 80). Generation of single-domain antibodies from naïve libraries, or amplification of the VNAR or VHH repertoire, results in lower affinity and broader specificity to antigens than immunized animals (81). Antibody production and validation are extremely time and money-consuming processes, especially for ND-specific research. Significant advancements have been made in developing synthetic immunogenic molecules or chemical antibodies that leverage short nucleic acid sequences for antigen detection to overcome time and expense issues (10, 11).

## 4 Aptamers

Aptamers are high-affinity nucleic acid ligands genetically scaffolded by selecting functional RNA molecules into short sequences of oligonucleotides *in vitro*, termed SELEX (10, 11). Approximately 5-15 kDa, aptamers consist of 20 to 100 nucleic acid residues and can be generated to target a particular region of interest rather than an entire molecule. Aptamers have many beneficial characteristics that mirror single-domain antibodies, including specificity, biophysical stability, and small size (82). Furthermore, aptamers can return to their native state after being denatured, making them advantageous tools for diagnostic and therapeutic applications. Unlike mAb manufacturing, aptamer production is not prone to contamination, is highly economical, and is easily scalable in that no model organisms or cell lines are required (83).

Both Ig isotypes in camelids and cartilaginous fish lack light chains, reducing their size to around 1/10th of mAbs (3, 72). Aptamers can be as small as 1/15th of mAbs, enabling potential BBB passage by size and solubility (83). Their small size allows high biodistribution within the body and intracellular robustness, but it also means rapid renal clearance, presenting a significant problem for chronic disorders like NDs. However, a simple solution is fusion to the Fc-region, allowing FcRn-mediated recycling in endothelial cells along the BBB (84).

Despite highly advantageous qualities, clinical ND trials for Nbs and aptamers are currently limited (85, 86). Unmodified aptamers that have progressed to clinical trials generally led to poor results from rapid clearance and patient instability (87). As there have been well-documented strategies to bypass this issue, including albumin, polymeric materials, and conjugation to the Fc domain, researchers should thoroughly assess the aptamer’s biophysical dynamics (88, 89). Despite this, there are concerns about how viable and safe these non-humanized models will work in patients, with some clinical trials being pulled due to FDA concerns (38). Further concerns have been expressed that aptamers’ ability to produce wanted outcomes in humanized models relies on the model they were created in and can result in unwanted neurotoxicity from aptamers interacting with complexes and proteins not previously addressed (90, 91).

## 5 Immunodiagnostic and therapeutic targets

Gold standard techniques involve labor-intensive animal models and immunodiagnostics restricted to postmortem tissues, presenting comparable barriers between NDs. The prion field has significantly advanced the functionality of transgenic (Tg) mice and medical translation to recapitulate natural ND progression (92–94). However, other NDs that involve heterogeneous protein species, i.e., tau and ɑ-synuclein, lack models and prion strains with essential characteristics of clinical disease presentation. While this presents many problems, reliance on pre-formed fibrils (PFFs) that do not mimic human tau or ɑ-synuclein prion strains has resulted in variable therapeutic efficacy once translated to human trials. Moreover, available anti-amyloid therapeutics are expensive and lack beneficial evidence, likely correlated with variance from Tg models and PFFs that do not faithfully recapitulate natural human disease (95, 96).

Current federally approved human and animal diagnostics are limited by the lower sensitivity of mAbs, requiring tissues with high concentrations of target proteins confined to the CNS and impeding early detection opportunities (97, 98). The hindrance of postmortem samples has resulted in vastly intricate, costly, and time-consuming differential diagnosis and symptom presentation methodologies (99). Despite decades of research focused on diagnostics, prevention, and therapeutics, 1 in 5 patients with dementia-like diseases are misdiagnosed, and approximately 20% are on inappropriate medications (100). As these conditions are substantially time-sensitive, with symptom presentation arising in the late stages of the disease, there is an increasing need for timelier and more specific antemortem diagnostics (101, 102).

**Figure 1:**
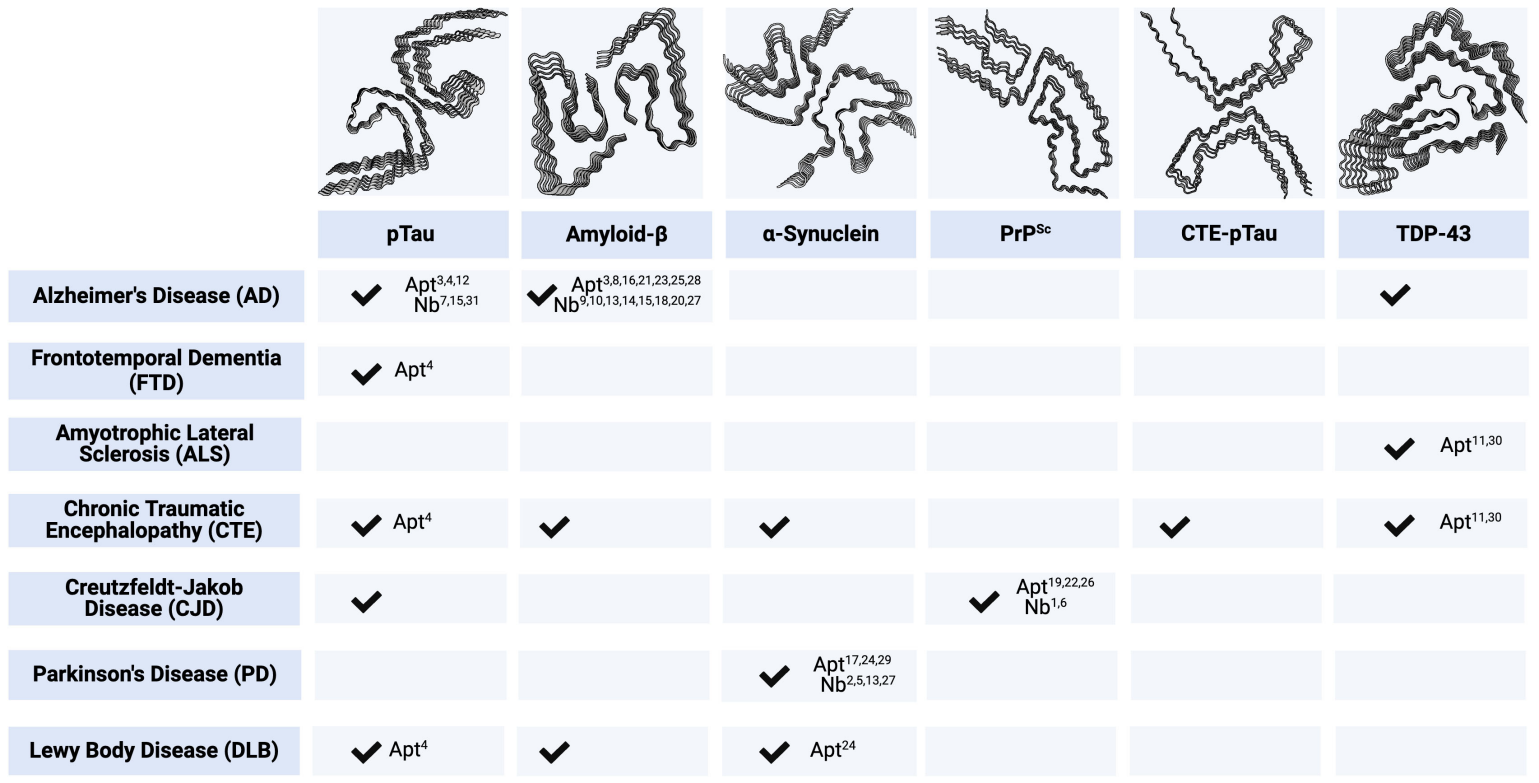
Key Immunodiagnostic and Therapeutic Targets by Associated Neurodegenerative Disease. Checkmarks indicate the presence of corresponding protein aggregates in respective ND. Numbered annotations correlate to candidate single-domain antibodies (Nbs) and aptamers (Apt), provided in **Supplementary Table S1**. All protein structures are human-derived except PrP^Sc^ are 263K prions from Tg7 mice. pTau PDB code - 5O3L (104). Amyloid-β PDB code - 5OQV (105). ɑ-Synuclein PDB code - 6H6B (106). PrP^Sc^ PDB code - 7LNA (107). CTE-pTau PDB code - 6NWP (108). TDP-43 PDB code - 7KWZ (109). Created with BioRender.com.

**Figure 2:**
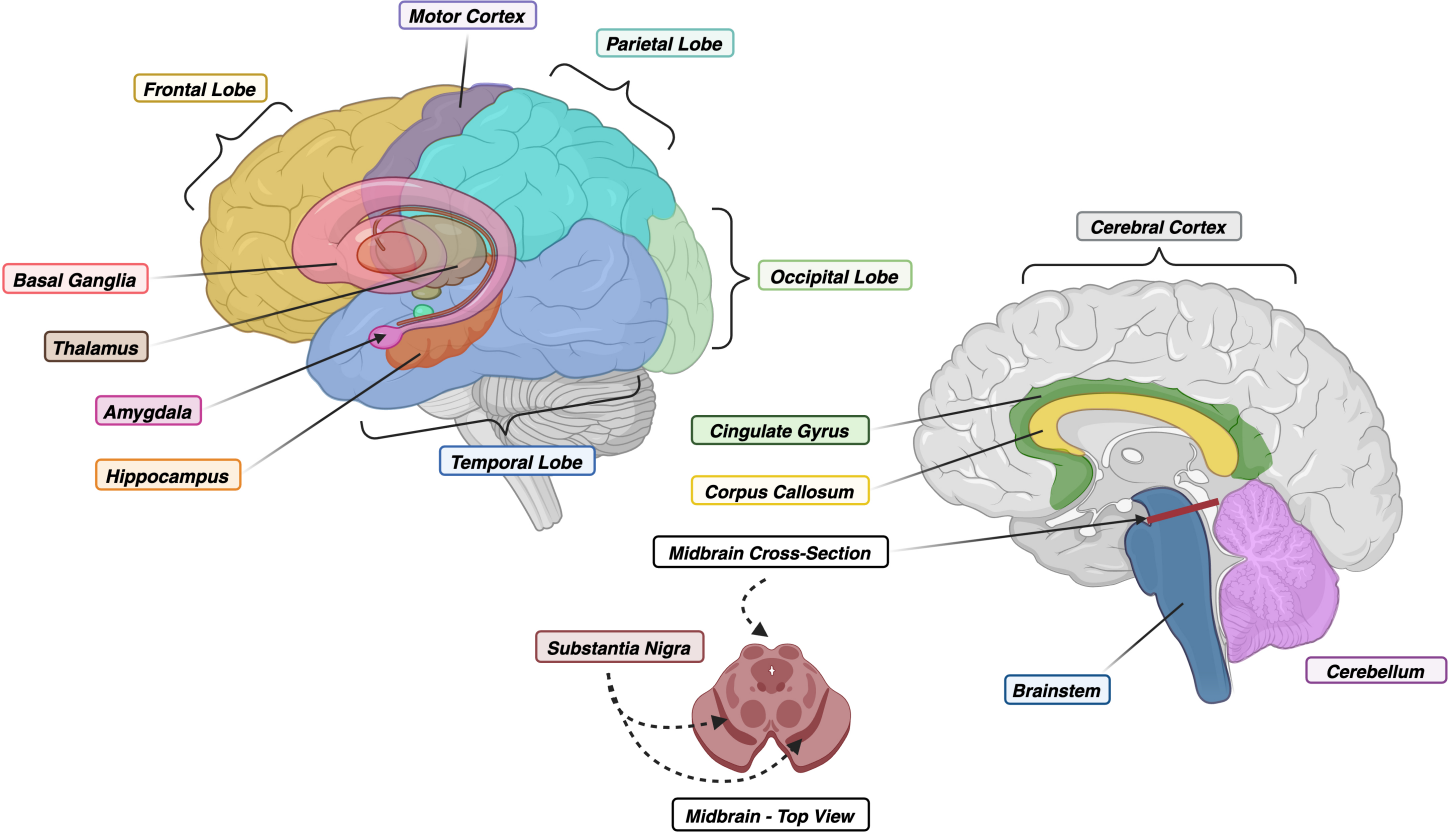
(*Left*) Lateral View of Human Brain with Floating Limbic and Gray Matter Structures. (*Right*) Midsagittal View of Human Brain with Cross-Section of Midbrain. Colors correspond to regions within **Figure 3**. Created with BioRender.com.

**Figure 3:**
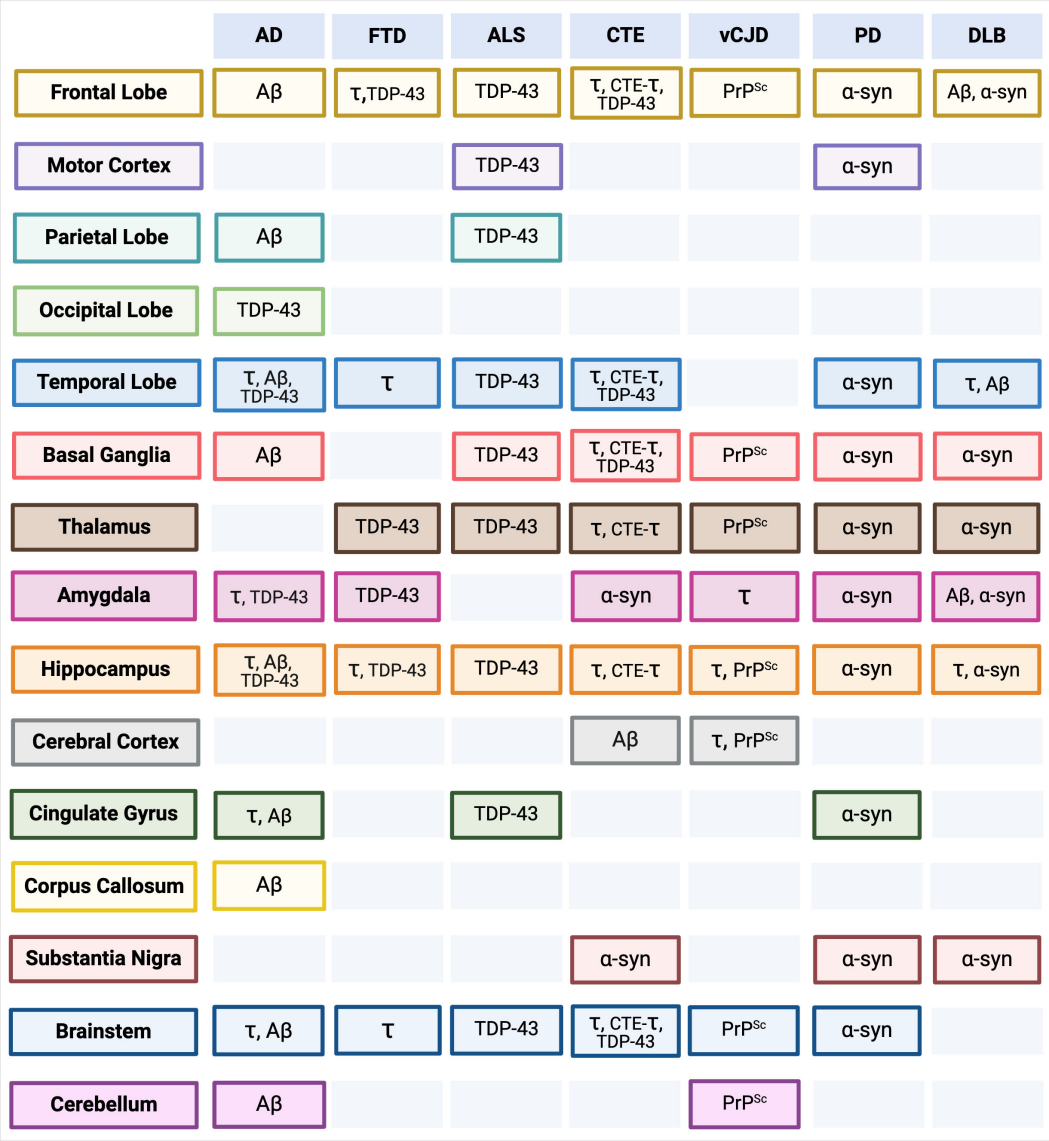
Anatomical Brain and Brainstem Regions by Neurodegenerative Disease (ND) (*Top Row*) and Misfolded Protein Aggregate Deposits (*see* **Figure 1**). Separate associated anatomical regions of each ND and misfolded protein aggregate deposits are visualized in **Supplementary Figure S1**. Alzheimer’s Disease (AD), Frontotemporal Dementia (FTD), Amyotrophic Lateral Sclerosis (ALS), Chronic Traumatic Encephalopathy (CTE), variant Creutzfeldt-Jakob Disease (vCJD), Parkinson’s Disease (PD), Lewy Body Disease (DLB). pTau (τ), Amyloid-β (Aβ), ɑ-Synuclein (ɑ-syn), CTE-pTau (CTE-τ) (110, 112–135). Created with BioRender.com.

Many NDs share massive neuronal loss as a feature of disease, leading to similar, non-specific clinical phenotypes that fail to elicit an immune response, making prevention, intervention, and treatment strategies nearly impossible. Prion-like self-templating propagation mechanisms have been identified in other NDs like Alzheimer’s Disease (AD), Frontotemporal Dementia (FTD), Chronic Traumatic Encephalopathy (CTE), Amyotrophic Lateral Sclerosis (ALS), Dementia with Lewy Bodies (DLB), and Parkinson’s Disease (PD) (103). The coexistence of various misfolded proteins and conditions has led to elusive and cloudy diagnoses, implying probable cross-over between NDs (**Figure 1**). Misfolding events for NDs typically occur in the neocortical or entorhinal cortices in the temporal lobe, resulting in co-conditions from probable cross-seeding events (110, 111) (**Figures 2**, **3**, **Supplementary Figure S1**).

Diagnostic and therapeutic strategies for NDs based on traditional approaches have largely failed to provide healthcare breakthroughs. In light of recent advancements in nanobody and aptamer research and development, we will review key protein targets classified by major CNS protein deposits observed in NDs (reviewed in 136–142). The following proteins dually represent biomarkers and therapeutic targets of NDs (**Figure 1** and **Supplementary Table S1**).

### 5.1 The mammalian prion protein in prion diseases

Prion Diseases, or Transmissible Spongiform Encephalopathies (TSEs), are rapidly progressive neurodegenerative diseases of mammals with a 100% fatality rate. TSEs of global importance include Creutzfeldt-Jakob Disease (CJD) in humans, CWD in cervids, and Bovine Spongiform Encephalopathy (BSE) in cattle (143). The pathogenic agent associated with TSEs derives from the highly conserved major mammalian prion protein (PrP) expressed by the *PRNP* gene. PrP^C^ is a glycophosphatidylinositol anchored protein that is abundantly expressed on the outer membrane of cells within the CNS and immune system (144, 145). The normal PrP^C^ isoform comprises an unstructured N-terminus, a conserved middle region, and a globular C-domain rich in alpha helices and minimal beta-sheet content (146, 147). The pathogenic PrP^Sc^ isoform lacks alpha-helical structures but is rich with high beta-sheet content primarily constituted in the hydrophobic core (148). Prion strain diversity has created a spectrum of clinical presentations and pathologic features, i.e., variant CJD (vCJD) is highly distinctive compared to classical or sporadic CJD (sCJD) and other NDs (149). The median age onset for vCJD is late 20s, with long incubation periods and early presentation of psychiatric symptoms, whereas sCJD has a median age onset of 58 years old, a shorter clinical duration, and early onset of neurological signs (112).

The generation of PrP-specific antibodies in the late 1990s into the 2000s identified two concerns. First, there was an inability to create mAbs specific to the infectious PrP^Sc^ isoform, which resulted in a wide variety of mAbs seeking different epitopes using *Prnp^0/0^* mice (150, 151). Second, an influx of mAbs induced neurotoxicity in PrP^Sc^-infected animal models associated with the flexible tail of the N-terminus (FTgpi-expressing and tga20 mice) (152, 153). Currently, PRN100 is the only anti-PrP^C^ mAb to reach human clinical trials, where signs of PrP^Sc^ clearance were potentially attributed to the mAb treatment. Abnormalities in white cell counts, protein levels, and postmortem neuropathology findings were ascribed to the rapid clearance of PrP deposits. However, three patients developed bacterial infections during treatment, resulting in the death of two patients. Potentially adverse findings, insufficient data, and clinical significance suggest the need to identify mAb-associated neuro or immunotoxic complications and alternative drug delivery models (57, 154).

Various single-domain antibodies have been generated to target PrP, including VHH nanobodies, Nb484, and PrioV3. *In vitro* protein crystallization of human PrP in complex with Nb484 inhibited PrP^Sc^ propagation by competitively binding the hydrophobic region of the infectious PrP isoform (155). These data unequivocally pointed toward the role of the hydrophobic region in PrP^Sc^ formation and provided the first documentation of an epitope associated with infectivity (4). PrioV3 exhibited migration across the BBB and negated prion replication in mouse neuroblastoma cell lines (156). Other PrioV3 studies using FVB/N mice likely leads to prion infection tolerance due to the PRNP species barrier between mice and humans (157, 158). Furthermore, camelids express the highly conserved mammalian prion protein; thus, VHHs are likely to develop tolerance to PrP^Sc^ (3). The lack of follow-up studies and preferential binding to the c-terminus in both isoforms suggests PrioV3 might be more suited for diagnostic applications.

RNA-based PrP aptamers or synthetic nucleic acid ligands have been produced specific to infectious isoform PrP^Sc^ *in vitro*, including DP7, that reduces PrP^Sc^ production in 3F4-ScN2a cell lines, SAF-93 primarily utilized in sandwich SPR detection assays, and R24 that displays inhibition to the conversion of infectious PrP (159–161). Of these, R24 is an unimolecular structure of two G-quadruplex R12 aptamers that bind conjointly, allowing stability upon interaction with prion-infected GT1-7 cells to block the infectious conversion of PrP (161). The g-quadruplex structure binds to PrP similarly to mRNA, allowing insight into how messenger RNA might be involved with protein misfolding (162). R24 displayed one of the lowest recorded IC_50_ values and highest anti-prion ability to date within prion-infected cell based assays among all anti-prion materials (Nbs, RNA/DNA aptamers, etc.) at 100 nM, providing a novel inhibition mechanism and insight into prion pathology and potential therapeutics (139, 161).

Despite the lack of clinical trials for potential therapeutics in prion disease, prion research insights have informed the entire ND field. Molecules specific to infectious PrP^Sc^, such as VHH Nb484 and RNA-based aptamer R24, represent significant advancements given that both display the ability to inhibit PrP^C^ to PrP^Sc^ conversion *in vitro*. These studies have the potential to inform future therapeutics.

### 5.2 Amyloid-beta in Alzheimer’s disease

Alzheimer’s Disease (AD) is the most common age-associated ND characterized by dementia, cognitive decline, and synaptopathy (110). Although numerous mechanisms have been proposed for the origins of AD, the majority of research focuses on the amyloid hypothesis, which states the misfolding of extracellular beta-amyloid (Aβ) aggregates into plaques causes neurodegeneration and, therefore, is the hallmark of AD (163). The hypothesis identified Aβ as the causative agent for AD; however, key AD neuropathologies include hyperphosphorylated tau NFTs (164, 165).

Maintenance of Aβ involves cleavage of the amyloid precursor protein (APP), and improper splicing gives rise to neurotoxic products that induce neurological dysfunction and the formation of fibrils (166, 167). Monomers of Aβ are soluble, presenting an opportunity to create antibodies; however, the misfolding of peptides results in the formation of multiple neurotoxic Aβ peptide species (i.e., oligomers, protofibrils, fibrils) with non-conventional epitopes. Current mAbs only target the healthy Aβ peptides and are unable to target all neurotoxic Aβ species in AD patients, preventing successful and reliable detection or potential therapeutics (168–170).

Monoclonal anti-amyloid drugs (i.e., Donanemab, Aducanumab, Lecanemab) have consistently progressed through human clinical trials without adequate assessment of their impact on the BBB’s structural integrity or potential adverse reactions (171–173). Donanemab, approved by the FDA in 2024, is a mAb specific to the post-translationally modified N-terminal truncated form of Aβ and is thereby selective to amyloid plaques. Early studies indicate that Donanemab reduces amyloid plaque burden with a slowing of cognitive decline in early-stage Alzheimer’s patients. However, targeting insoluble Aβ peptide species through several previously FDA-approved mAb therapeutics has thus far yielded limited improvements in patient quality of life and the spectrum of neurodegenerative disease-associated deficits. The lack of clarity in the current definition of clinical efficiency, and potential for emerging neurotoxic complications when using mAb-based therapeutics, underscores the need to better define these traits for ND-associated immunotherapeutics (52, 171, 174, 175).

Single-domain antibodies have been found to have the selectivity for the neurotoxic oligomeric peptides without negative consequences; however, there has been no progression to clinical trials (176). Multiple VHHs have facilitated the detection of the various neurotoxic Aβ peptide species, like VHH B10. Binding confirmation of VHH B10 *in vitro* to mature fibrils and protofibrils, with the selective avoidance of soluble or nontoxic peptides, monomers, and oligomers, is highly encouraging. However, VHH B10 could not reverse preformed fibrils and weakly bound to dissolved or disaggregated Aβ peptide and oligomers (177). Other single-domain antibodies that selectively bind to monomers and small oligomers have been applicable as detection tools (178). Another research group developed VHHs specific to Aβ by incorporating peptide segments into the variable domains within CDRs, allowing a bridge for overcoming low substoichiometric concentrations in samples outside the CNS (6). Follow up studies used this technique to produce a pool of VHHs with grafted motifs of amyloidogenic peptides that eliminate antibody cross-reactivity to other amyloidgenic proteins and neutralize toxicity in various Aβ conformers *in vitro* (179). Recent VHH studies are instead targeting soluble Aβ oligomers (SAβOs), given evidence of SAβOs serving as an intermediate between monomers and neurotoxic Aβ peptide species. Investigative work of VHH Nb E3 selectively binding to discontinuous epitopes on SAβOs and Aβ plaques within AD-specific Tg(5xFAD) mice. VHH E3 bound to SAβOs and Aβ plaques are characteristically and spatially distinct within AD mouse models, providing evidence of E3’s capacity for early AD detection and potential therapeutic applications (180).

Aptamers have allowed highly sensitive detection of early-onset Alzheimer’s (181), while others have displayed inhibitory effects with high sensitivity but low selectivity (182, 183). BACE1 is a B-secretase that is elevated in AD due to being the key facilitating factor behind the cleavage of the APP (184). RNA aptamers S10 and TH14 have been developed to interact with the short cytoplasmic tail of the BACE1 *in vitro* (185). Generation of more aptamers modulated and inhibited BACE1 activity, and lowered Aβ concentrations in cell-free and M17 neuroblastoma cell models (186, 187). Moreover, VHHs specific to BACE1 have also been of therapeutic interest, leveraging adeno-associated virus-based vectors for BBB crossing. Anti-BACE1 VHH-B9 demonstrates a high affinity for BACE1 and potential pre-clinical efficiency in *APP^NL-G-F^* mouse models, given a single dose relieved AD cognitive deficits and Aβ pathology over 12 months (188).

While the dropout rate of mAb therapeutics in AD clinical trials continues to grow, such studies remain valuable because they provide critical baseline data for scientists to consider. mAb treatments targeting soluble Aβ neurotoxic species, such as Lecanemab, show ARIA (amyloid-related imaging abnormalities) scores comparable to placebo and alleviate cognitive decline, albeit with modest efficiency (175). Haynes et al., 2024 provide further evidence that small molecules targeting soluble neurotoxic peptides in neurodegenerative diseases could facilitate higher clinical efficacy and improve the quality of life in early-stage neurodegenerative disease (188). Advancements using grafted amyloid motifs into VHH gammabodies increase specificity and reduce toxicity, providing a method for single-domain antibodies specific to other ND proteins. On the other hand, aptamers and VHHs that target BACE1 inhibit interaction and the cleavage of APP, displaying therapeutic potential and, at the same time, the importance of identifying non-amyloid targets.

### 5.3 Tauopathies and other associated proteinopathies

The microtubule-associated protein tau is a family of naturally soluble proteins that promote microtubule assembly expressed by the *MAPT* gene in the human brain (189). Abnormal or hyperphosphorylation of tau (pTau) self-assembled into paired-helical filaments (PHF-tau) and neurofibrillary tangles (NFTs), result in the pathology behind many tauopathies, including AD (190, 191). Six tau isoforms are present within the brain by alternative splicing in the *MAPT* gene. Differences in isoforms include the presence or absence of two N-terminal inserts and three or four imperfect repeat regions in the C-terminus within the microtubule-binding domain (192). Variations of pTau have clinical and neuropathological heterogeneity in other tauopathies like FTD, CTE, and DLB. The wide array of NDs containing pTau underlines the idea of cross-seeding into comorbid neuropathological conditions and conditions not previously considered to involve pTau (113). Examples include subtypes of ALS, PD, and the use of CSF pTau levels as a biomarker for CJD (114, 193, 194).

CTE is one of the greatest mysteries within ND research, characterized as progressive neurodegeneration from mild traumatic brain injuries (195, 196). In a study of neuropathologically confirmed CTE cases, ~35% had additional comorbid neuropathological findings, including Aβ plaques, Lewy Bodies, and 43kDa TAR DNA-binding protein (TDP-43) inclusions (115, 197). The vast diversity of clinical and pathological signs pointed toward a novel CTE-specific scaffold distinct from those in AD patients (108, 198). Heterogeneity within the spectra of FTD and ALS patients presents similar issues with clinical and neuropathological variability (116, 117) (**Figures 1**, **3**).

Heterogeneity in pTau inclusions has presented difficulties in discerning what epitopes are significant for diagnostics and potential therapeutics (199). One of the antibodies routinely used for pTau detection is PHF-tau-specific mAb AT8, despite its general instability (200). Additional anti-tau mAbs targeted the N-terminus of extracellular tau, but phase II human clinical trials resulted in insignificant findings (201–203). On the other end of the spectra, recent mAb studies selected the C-terminus of TDP-43 as an epitope, displaying decreased aggregation and neurotoxic effects of intracellular inclusions. Targeting the C-terminus allowed clearance of misfolded aggregates and avoided RNA recognition motifs associated with neurotoxicity and neuron loss in ALS (204).

Antibody fragments have facilitated the detection of the various isoforms and species of pTau with multiple VHHs developed against pTau. Discovery work exploring BBB-permeable probes has allowed the development of VHHs to label amyloid deposits and NFTs (7). Tau-specific VHH PrioAD120 was created by immunization of alpacas using AD samples; however, it is one of two Nbs without brain diffusion results in FVB/N mice (157). Recent studies identified a Tau VHH-Fc fusion protein that selectively binds to fibrillar Tau aggregates rather than soluble Tau monomers. τ-Nb-Fc fusion proteins display low non-specific binding and high specificity comparable to FDA-approved anti-τ mAb Elotuzumab *in vitro* using P301S Tg and wild-type (WT) mice and human brain samples. The tau conformational nanobody also demonstrates higher biophysical stability and specificity than conformational mAbs currently employed in clinical use (9).

Explorative studies using single-stranded DNA to identify tau-specific aptamers displayed that tau can bind to both ssDNA and double-stranded DNA. High-affinity and specificity tau-ssDNA interactions are sequence specific and promote dsDNA dissociation, suggesting ssDNA aptamers may be ideal candidates in tau detection and treatments (205). This has led to a wave of highly specific ssDNA aptamers able to detect, inhibit, against, and protect the brain from the neurotoxic impacts of pTau (140, 206, 207). Furthermore, aptamers have been used to describe RNA sequences among comorbid NDs like ALS and FTD patients utilizing an RNA aptamer against full-length TDP-43 (208). Continued studies using this aptamer uncovered mechanism insights of RNA sequences shown to destroy aggregation of TDP-43 *in vitro* (209).

The increased prevalence of comorbidity and potential for protein cross-seeding is widely recorded in tauopathies and underscores the need to address more than one protein target in a candidate therapeutic (**Figures 1**, **3**). VHH τ-Nb-Fc fusion proteins reveal how single-domain antibodies can overcome reactivity, specificity, and stability limitations in drug delivery applications. Moreover, using aptamers as discovery molecules has shed light on ssDNA interactions with tau and RNA sequences that can be used to dissociate TDP-43 aggregates.

### 5.4 Synucleinopathies

Hallmark clinical presentation of Dementia with Lewy Bodies (DLB) is intracellular fibrillar Lewy bodies composed of α-synuclein (α-syn) or Lewy neurite aggregates causing loss of dopaminergic neurons resulting in severe motor impairment. Core clinical features are variations in cognitive function, particularly in attention and alertness (118), as well as inconsistencies in behavior and hallucination. Parkinson’s disease (PD) is attributed to specific movement clinical signs called parkinsonism, which defines bradykinesia with hypertonia, spasticity, and tremors. However, DLB can resemble AD, and it can be challenging to differentiate between PD in clinical scenarios due to pathogenic heterogeneity (**Figure 3**). DLB typically has minimal dopaminergic uptake in the caudate and putamen compared to AD and healthy patients and retention of the medial temporal lobe, resulting in diagnosis primarily from distinguishable clinical signs and associated patterns in atrophy (210).

The intracellular nature of Lewy neurites has proposed a tricky hurdle for detection, disaggregation, clearance, and potential therapeutics. Synuclein studies heavily rely on PFFs in WT mice due to the inability to produce human synuclein prion pathology in DLB models. Other models using oligodendrocyte-specific promoters to express WT human α-syn (MBP1, PDGF, CNP mice) develop pathology over varied time points (211). While these models can clarify α-syn function, consequential impacts on early development hinder clinical relevance in therapeutic applications (212). Structural differences in PFFs to natural strains induce distinct neurobiological properties, and synthetic PFFs fail to recapitulate natural human disease (213, 214). Despite this, Nbs and aptamers have identified novel α-syn mechanisms, diagnostics, and potential therapeutic targets.

Researchers leveraged nanobody libraries to create VHHs specific for α-syn, including grafting various monomers into VHH sequences that display neutralization potency and reduce α-syn aggregation *in vitro* (8). Despite the inability to slow fibril formation, NMR spectrometry studies of NbSyn2 displays fibril interaction without inducing structural changes via binding to the last four residues within the C-terminus (8, 215). Other VHHs fused to a proteasomal targeting PEST motif increased solubility and clearance of α-syn using multiple cell culture models *in situ*, reducing the toxicity of aggregates (216). Further *in vivo* studies using VHHs-PEST exhibited increased clearance in Sprague-Dawley rats by targeting α-syn monomers but did not abrogate prion spreading and neurotoxicity (217). VHH PFFNB2 prevented neurotoxicity and prion spreading by selective targeting of α-syn fibrils in HEK293T cell lines and α-syn Tg mice, PAC-Tg(SNCAWT), advancing the therapeutic development of PD (5). Ossinax, a biotechnology company, aims to facilitate drug transport across the BBB by a VNAR specific to transferrin receptor 1 (TfR1) prevalent on brain endothelial cells. Fusion of VNAR TXB4 to a TrkB agonist antibody, a therapeutic target for PD, enabled brain protection leveraging the VNAR TXB4-TfR1 brain transport shuttle in BLAB/c mice (218).

Aptamer development began with engineered single-chain Fv antibody VH14 developed to target the non-amyloid component of the monomeric α-syn. While having the highest affinity for its target, it did not prevent cytotoxicity *in vitro* (219). Further aptamer exploration of M5-15 targeting both α-syn monomers and oligomers with higher binding affinity to oligomers allows a highly sensitive method for the detection of PD (220, 221). DNA aptamers F5R1 and F5R inhibited aggregation *in vitro* and suppressed oxidative stress associated with Lewy neurites in mitochondria (222, 223). Later studies showed their ability to rescue Lewy neurites and reduce aggregates, improving motor function in mouse models (224). Increased drug efficacy studies aiming to alleviate Parkinsonism symptoms continue to highlight the need for models and prion strains that faithfully recapitulate natural disease progression and presentation. Single-domain antibodies have facilitated promising findings, including VHH PFFNB2 preventing prion spreading by selective targeting of α-syn fibrils and VNARs that shuttle agonist antibodies across the BBB. Finally, aptamers have provided insights into neuropathological mechanisms of synucleinopathies while addressing other possible cytotoxic impacts of Lewy neurites when targeting aggregates.

## 6 Conclusion

Significant limitations surround traditional mAb-based diagnostic and therapeutic approaches to NDs. When considering potential immunodiagnostic and therapeutic protein targets, several NDs involve multiple endogenous misfolded proteins (**Figure 1**), with a growing body of evidence suggesting that cross-seeding of protein species plays a role in disease progression (**Figures 2**, **3**). This observation indicates that future breakthroughs in the detection and prevention of ND must identify key protein biomarkers during the earliest stages of disease and mechanisms to prevent protein misfolding cascades. Single-domain antibodies and aptamers can bind to epitopes hidden away or concealed within misfolded forms and protect the structural integrity of the BBB. These molecules can be employed together, creating a powerful diagnostic tool for multifaceted conditions while addressing more than one protein. We posit that single-domain antibodies and aptamers have great potential to address current gaps in ND diagnostics and therapeutics.

## Supplementary material

Supplementary Table 1 Candidate Single Domain Antibodies (Nb) and Aptamers (Apt) for ND Immunodiagnostic and Therapeutic Targets. Numbers correlate to annotations in **Figure 1**. ssDNA: single-stranded DNA, scFv: single-chain variable Fragment.

Supplementary Figure 1 Associated Brain Anatomical Regions by Neurodegenerative Disease (*Left of red line*) and Misfolded Protein Aggregate Deposits (*Right of red line*). Composite associated brain anatomical regions by both neurodegenerative disease and misfolded protein aggregate deposits are visualized in **Figure 3**. Created with BioRender.com.
