# Supplementary figures and images for: Single-domain antibodies and aptamers drive new opportunities for neurodegenerative disease research
Source: Front Immunol. 2024 Aug 22;15:1426656. doi: 10.3389/fimmu.2024.1426656 (PMC11374656; doi:10.3389/fimmu.2024.1426656)

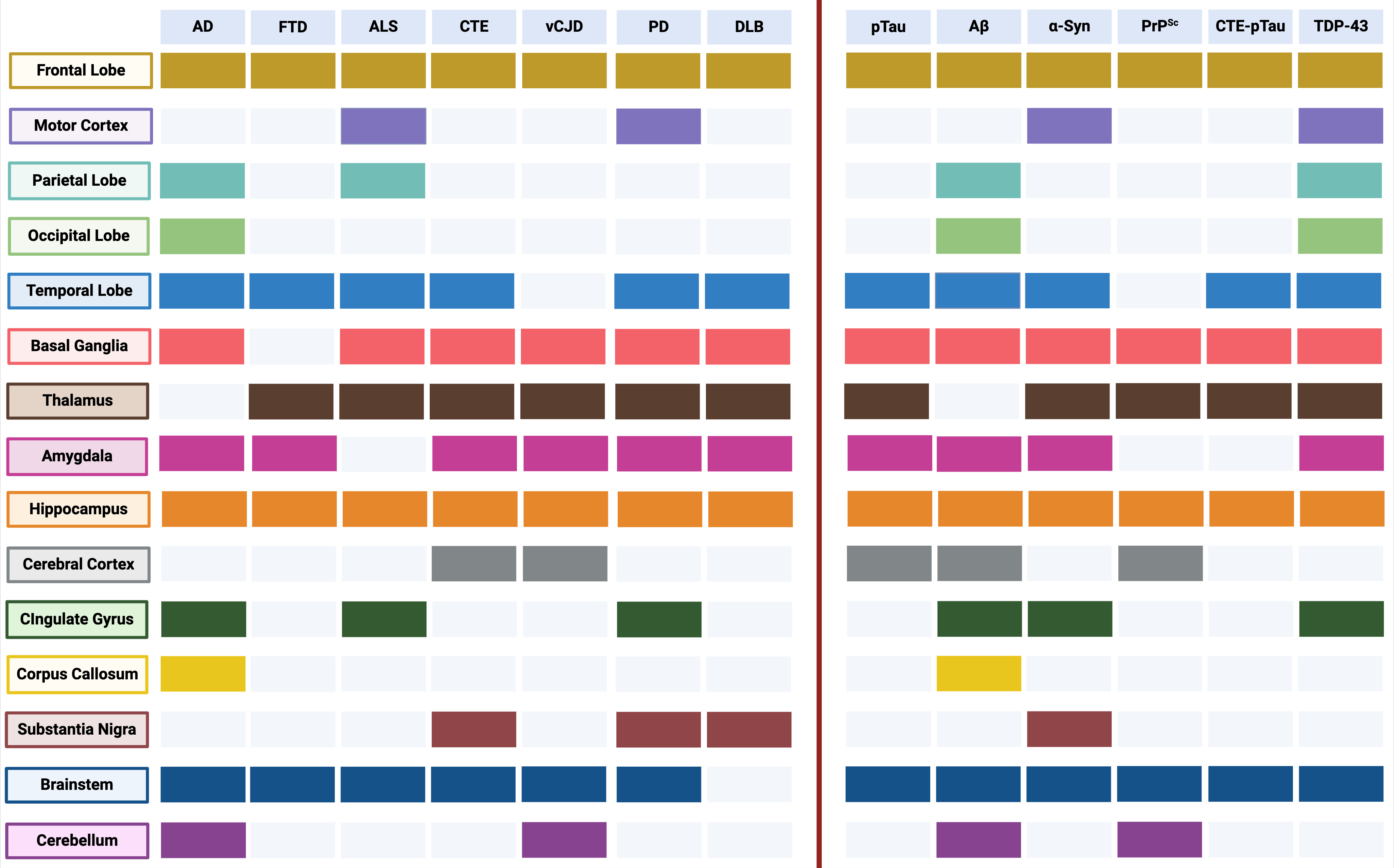

Supplement: Supplementary Figure 1 — Associated Brain Anatomical Regions by Neurodegenerative Disease (Left of red line) and Misfolded Protein Aggregate Deposits (Right of red line). Composite associated brain anatomical regions by both neurodegenerative disease and misfolded protein aggregate deposits are visualized in Figure 3 . Created with BioRender.com. [file Image1.jpeg]
